# Whole Transcriptome Sequencing Analyzes the Interactions of mRNAs and ncRNAs in Cholangiocarcinoma

**DOI:** 10.1002/cam4.70906

**Published:** 2025-04-30

**Authors:** Qinlei Wang, Menshou Chen, Jingru Zhang, Chuan Feng, Haoran Li, Jingyu Guo, Zhaowei Sun, Yujie Feng

**Affiliations:** ^1^ Department of Hepatobiliary and Pancreatic Surgery The Affiliated Hospital of Qingdao University Qingdao Shandong China; ^2^ Department of Medicine Qingdao University Qingdao Shandong China

**Keywords:** ceRNA network, cholangiocarcinoma, enrichment analysis, potential biomarkers, whole‐transcriptome sequencing

## Abstract

**Background:**

Cholangiocarcinoma is a common hepatic malignant tumor with an unfavorable prognosis. Therefore, we systematically evaluated the transcriptomic landscape of CHOL by whole transcriptome sequencing technology in this study and constructed a ceRNA network associated with CHOL.

**Methods:**

First, whole transcriptome sequencing between the tumor tissues of CHOL and adjacent cancer tissues adjacent to the tumors from six patients with CHOL was performed. Then, a differential expression analysis between the CHOL group and adjacent cancer group was performed to screen significant markers. Subsequently, target gene predictive analysis and co‐expression analysis were implemented to construct a ceRNA and protein–protein interaction network in CHOL, and enrichment analysis was performed to investigate gene‐related molecular pathways.

**Results:**

The results showed that there were 761 differentially expressed mRNAs, 47 differentially expressed miRNAs, 61 differentially expressed lncRNAs, and 1481 differentially expressed circRNAs in the adjacent cancer group compared with the CHOL group, respectively. Enrichment analysis of differentially expressed mRNAs showed that the PI3K‐Akt, calcium, and MAPK signaling pathways were significantly enriched. Hsa‐miR‐196b‐5p can be a sponge to adsorb lncRNA H19 and 101 downregulated mRNAs, constructing an lncRNA‐miRNA‐mRNA network. Hsa_circ_0025636, hsa_circ_0057335, hsa‐miR‐96‐5p, and hsa‐miR‐196b‐5p were involved in the circRNA‐miRNA‐mRNA network. Moreover, five core genes were obtained through PPI interaction analysis, which also played an important role in the ceRNA network.

**Conclusions:**

This study systematically presents a transcriptomic landscape of CHOL and identifies lncRNA/circRNA‐associated ceRNA networks that could provide insights for future treatment and prognosis of CHOL, laying a certain foundation for the study of molecular mechanisms and providing novel ideas for its prognosis and treatment.

## 1 Introduction

Cholangiocarcinoma (CHOL) is the second most common primary liver malignant tumor that originated from intrahepatic or extrahepatic bile duct epithelial cells [1, 2]. The average incidence of CHOL is low but gradually increasing from Asia to North America and Europe [3]. CHOL presents difficulties in the early diagnosis and treatment [4]. Coupled with the poor prognosis and the strong tendency for recurrence of CHOL [5], surgical resection and chemotherapy cannot significantly prolong long‐term survival. Therefore, early detection of CHOL is critical to provide patients with curative treatment and maximize clinical outcomes [6]. At present, although genomics and transcriptomics have significantly expanded our cognition of CHOL [7, 8, 9], its molecular pathogenesis is still unclear [10]. It is essential to understand the molecular pathogenesis and find prognostic biomarkers that may guide the clinical diagnosis and treatment.

Previous studies have focused on the role of genetic, epigenetic, and transcriptomic changes in tumor suppressor genes and oncogenes in the pathogenesis of CHOL. Sia et al. [11] used comprehensive molecular analysis to identify proliferative and inflammatory classes of intrahepatic cholangiocarcinoma. Mishra et al. [12] proved the importance of the Hippo signal in cholangiocarcinoma based on methylation data. Based on the RNA‐sequencing data from the TCGA database, Zhou et al. [13] identified differentially expressed mRNA, lncRNAs, and miRNA to construct ceRNA networks and determined that lncRNA HULC accelerated liver cancer by inhibiting PTEN in cooperation with miR15a autophagy. Long et al. [14] used differentially expressed genes between cholangiocarcinoma and adjacent liver to construct ceRNA networks mediated by lncRNA imbalance. They also found that the low expression of LINC00261 was significantly related to TNM stage, lymph node status, and distant metastasis. A study based on whole transcriptome sequencing data of 8 cases of cholangiocarcinoma found the potential role of miR‐144‐3p in the pathogenesis of CHOL, but the data was not shared [15]. Although previous studies have made some progress in revealing the molecular mechanisms of CHOL, such as exploring its epigenetic and transcriptomic changes, identifying different molecular subtypes and key signaling pathways, and constructing competing endogenous RNA (ceRNA) networks to elucidate gene regulatory mechanisms, a comprehensive understanding of the transcriptomic landscape of CHOL is still incomplete. In particular, current research on the role of long non‐coding RNA (lncRNA) and microRNA (miRNA) in the pathogenesis of CHOL and the function of ceRNA networks in regulating gene expression remains insufficient.

From the current research, the available transcriptome data of cholangiocarcinoma are very few, especially the whole transcriptome sequencing data. Most studies rely on the data in the open TCGA database (*n* = 45). Therefore, in this study, we sequenced the whole transcriptome of six patients with CHOL, screened the differentially expressed mRNA, miRNA, lncRNA, and circRNA, constructed the ceRNA network, and identified the potential crucial hub genes of the cancer mechanism. We also compared our results with the study by Chu et al. [15]. This study provides a new molecular basis for the occurrence and progress of CHOL and potential biomarkers for early diagnosis and treatment.

## 2 Materials and Methods

### 2.1 Patient Samples

Bile duct tumor tissues and corresponding adjacent tissues were collected from six male patients with cholangiocarcinoma (CHOL). All participants met the following inclusion criteria: (1) age ≥ 18 years, (2) cholangiocarcinoma confirmed by pathology, (3) no prior surgical, radiotherapeutic, or chemotherapeutic treatment, and (4) provision of informed consent. Patients with other malignancies, pregnant or lactating women, or those unwilling to participate were excluded. The median patient age was 65.0 (61.0–72.0) years (Table 1, Table S1). The study protocol was approved by the Medical Ethics Committee of Qingdao University (No. QYFYWZLL26664).

**TABLE 1:** Clinical characteristics of six patients with cholangiocarcinoma.

Characteristic	Patients (*n* = 6)
Gender	
Male	6 (100%)
Female	0
Median age (interquartile range), years	65.0 (61–72)
Smoking	
Yes	5 (83.3%)
No	1 (16.67)
Alcohol dependence	
Yes	3 (50.0%)
No	3 (50.0%)
Tumor stage (TNM)	
T1	3 (50.0%)
T2a/T2b	2 (33.3)
T4N1M1	1 (16.67)
Liver function index	Mean (range)
ALT	152.37 (24.4–310.0)
AST	76.42 (23.3–180.7)
Total bilirubin	206.45 (14.0–435.1)
Albumin	41.09 (35.33–47.1)
HBV surface antigen	0.03 (0–0.14)
AFP	4.21 (1.78–5.77)
CEA	4.3 (2.73–9.44)
CA125	103.63 (9.35–526)

### 2.2 RNA Extraction and Library Construction

Total RNA was extracted from tumor and adjacent tissues using TRIzol (Thermo Fisher Scientific) and assessed using a NanoDrop 2000 Spectrophotometer (Thermo Fisher Scientific) and Agilent 2100 Bioanalyzer (Agilent Technologies). Genomic DNA and ribosomal RNA were removed using DNase I and Ribo‐Zero rRNA Removal Kits (Epicenter). cDNA libraries were prepared with the TruSeq RNA Sample Prep Kit (Illumina) and sequenced on an Illumina HiSeq 2500 platform (125‐bp paired‐end reads). For miRNA analysis, libraries were constructed using the TruSeq Small RNA Library Prep Kit (Illumina) and sequenced on a HiSeq 2000 platform.

### 2.3 Differential Expression Analysis

Principal component analysis (PCA) was performed to identify outlier samples. Transcript expression levels (mRNAs, lncRNAs, miRNAs, circRNAs) were quantified as fragments per kilobase of transcript per million mapped reads (FPKM). Differentially expressed transcripts were identified using the DESeq2 package (version 1.32.0) with a threshold of log_2_(Fold Change) > 2 and *p* < 0.05.

### 2.4 Target Gene Prediction Co‐Expression, and Enrichment Analyses

Differentially expressed miRNAs (diff‐miRNAs) were analyzed in miRDB, miRTarBase 8.0, and TargetScanHuman 7.2 to identify candidate target genes. Differentially expressed lncRNAs (diff‐lncRNAs) and circRNAs (diff‐circRNAs) were examined in StarBase 2.0 for potential target genes. Pearson correlation analyses of diff‐mRNA/diff‐lncRNA and diff‐mRNA/diff‐circRNA pairs were then performed; pairs with *r* ^2^ > 0.9 and *p* < 0.05 were retained. GO and KEGG pathway analyses of differentially expressed mRNAs (diff‐mRNAs) were conducted using clusterProfiler (version 4.0.3), applying *p* < 0.05 and an enriched gene count > 3.

### 2.5 ceRNA Network Construction

Co‐expression results were integrated with target prediction data to identify miRNAs that concurrently regulate lncRNAs/circRNAs and mRNAs in an expression pattern opposite to the miRNAs. These triplets were visualized using Cytoscape (version 3.8.2) to construct lncRNA–miRNA–mRNA and circRNA–miRNA–mRNA networks.

### 2.6 Protein–Protein Interaction (PPI) Analysis

All differentially expressed genes were examined in the STRING database (version 11.5, minimum interaction score = 0.90) for high‐confidence protein interactions. The resulting PPI network was visualized with Cytoscape (version 3.9.1). Using the CytoNCA plug‐in (parameter “without weight”), node importance was evaluated by betweenness centrality (BC), degree centrality (DC), eigenvector centrality (EC), local average connectivity (LAC), and network centrality (NC).

### 2.7 Quantitative Real‐Time PCR (qRT‐PCR)

Total RNA was extracted using TRIzol, and cDNA was synthesized for qRT‐PCR on a Bio‐Rad CFX instrument. GAPDH served as an internal control, and relative expression was calculated by the 2^−ΔΔc^_t_ method. Primers used for lncRNAs and mRNAs are listed in Table S2. Statistical significance between two groups was defined as *p* < 0.05.

### 2.8 Cell Transfection

A cholangiocarcinoma (CHOL) cell line with the highest CAV1 expression was selected for transfection. Cells (1 × 10^6^/well in 6‐well plates) were seeded and incubated for 12 h before transfection with si‐NC or si‐CAV1 using Lipofectamine 2000. Knockdown efficiency was verified by qRT‐PCR. The target sequences were as follows: si‐NC: sense 5'‐UUCUCCGAACGUGUCACGUTT‐3', antisense 5'‐ACGUGACACGUUCGGAGAATT‐3', si‐CAV1: sense 5'‐CUGCGAUCCACUCUUUGAATT‐3', antisense 5'‐UUCAAAGAGUGGAUCGCAGTT‐3'.

### 2.9 Wound Healing Assay

Transfected cells at ~90% confluence were scratched with a pipette tip, washed with PBS to remove debris, and incubated in serum‐free medium. Images were captured at 0 and 24 h to evaluate the scratch healing rate.

### 2.10 Transwell Assay

Cells (2 × 10^4^) in serum‐free medium were placed in the upper chamber of a Transwell plate (with or without Matrigel for invasion assays); the lower chamber contained medium with 10% FBS. After 48 h, cells that migrated or invaded through the membrane were fixed, stained with crystal violet, and quantified under a fluorescence microscope.

### 2.11 EdU Assay

Cells (2 × 10^3^/well in 96‐well plates) were cultured for 24 h, then incubated with EdU for 2 h. After fixation and permeabilization, cells were stained and visualized under a fluorescence microscope; EdU‐positive cell percentages were recorded to assess proliferation.

### 2.12 TCGA CHOL Data Validation

Data from TCGA‐CHOL (36 tumor and 9 adjacent samples) were obtained through the Genomic Data Commons. Using DESeq2, we applied log_2_FoldChange > 2 and *p* < 0.05 to identify significant mRNA and miRNA changes and compared these to our study findings. The expression of core genes was further verified by unpaired Wilcoxon rank‐sum tests, with *p* < 0.05 considered significant.

## 3 Results

### 3.1 Differentially Expressed Coding and Non‐Coding RNA

**FIGURE 1:**
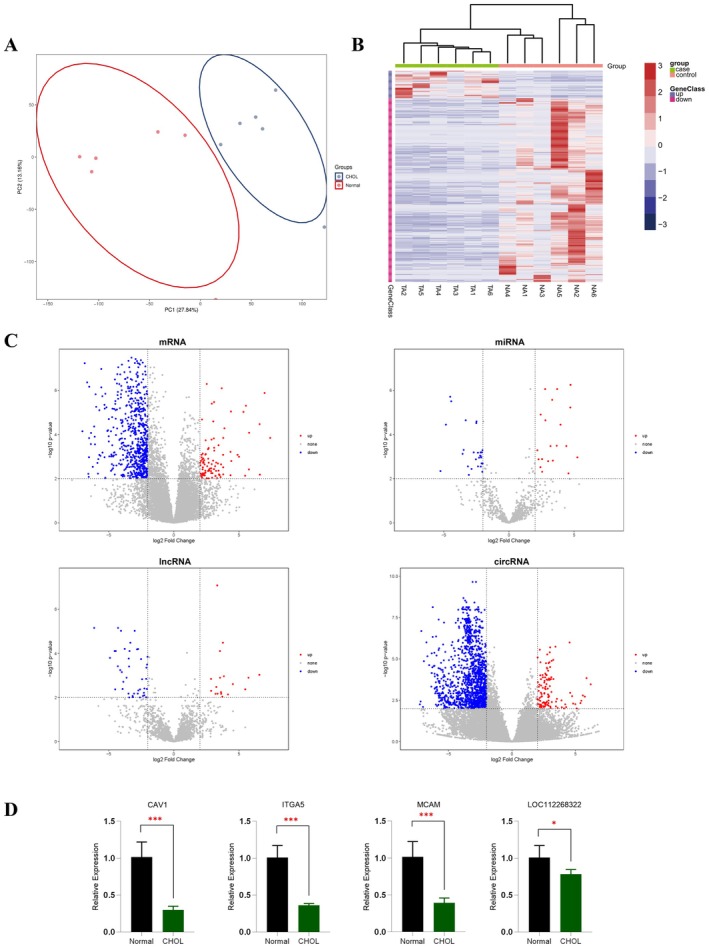
Differentially expressed RNAs. (A) Principal component analysis. (B) Heatmap of differential expression mRNAs. (C) Volcano plot of differentially expressed mRNAs, miRNAs, lncRNAs, and circRNAs. Red indicates upregulation, and blue indicates downregulation. (D) Validation of RNA expression by using qPCR. Data are presented as means ± SD (*n* = 6; *p < 0.033, **p < 0.002, ***p < 0.001).

Before analysis of differential gene expression, PCA was performed to verify dispersion between samples. The results showed that the samples in the different groups could be distinguished (Figure 1A). The clustering heatmap of differentially expressed genes of all samples showed that the cancer samples can be significantly separated from the adjacent cancer samples (Figure 1B). There were 61 diff‐lncRNAs (19 upregulation and 42 downregulation), 1481 diff‐circRNAs (110 upregulation and 1371 downregulation), 47 diff‐miRNAs (20 upregulation and 27 downregulation), and 761 diff‐mRNAs (99 upregulation and 662 downregulation) in adjacent cancer group compared with the CHOL group, respectively (Figure 1C). The upregulated and downregulated lncRNAs, circRNAs, miRNAs, and mRNAs are illustrated in Tables S3–S6. Furthermore, differentially expressed RNAs in this study were compared with similar studies. The results showed that only the diff‐miRNAs have no statistically significant difference compared to other non‐coding RNAs (diff‐mRNAs, diff‐lncRNAs, and diff‐circRNAs). The results are shown in Table S7 [15].

To confirm the differential expression level of RNAs in RNAseq experiments, randomly selected differentially expressed mRNAs and lncRNAs were analyzed and validated using qRT‐PCR. By validating genes through random selection, it ensures that the selected group of genes is more representative and can reflect the overall biological background, thereby reducing the result bias caused by selection bias [16, 17]. All selected mRNAs and lncRNAs were detected in 6 bile duct tumor tissues and 6 matched adjacent cancer tissues. The qPCR results were highly consistent with the RNAseq data. The results with statistically significant differential expression were exhibited in Figure 1D.

### 3.2 Enrichment Analysis of Diff‐mRNAs

**FIGURE 2:**
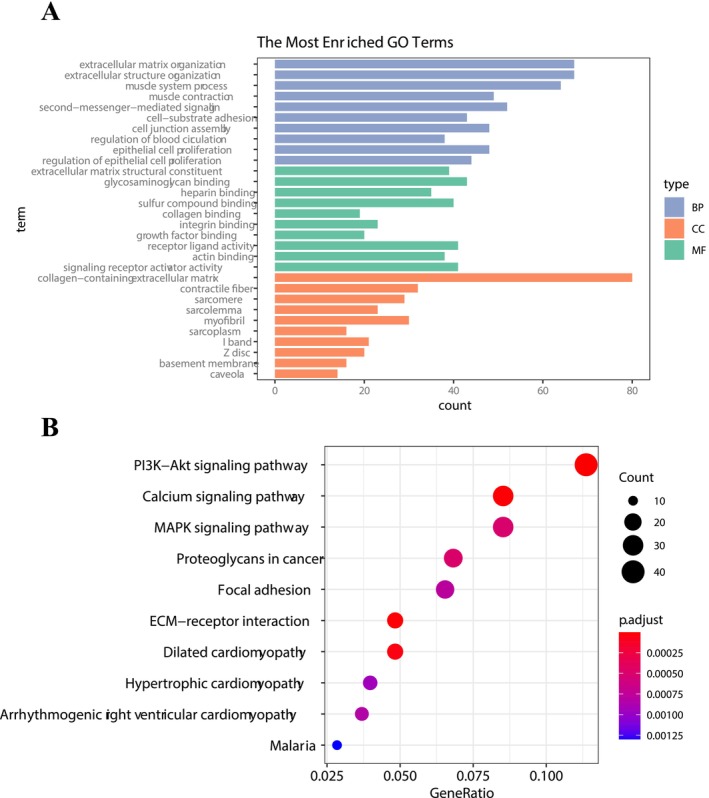
Enrichment of differential expression mRNAs. (A) Top10 GO terms enriched by differentially expressed mRNAs. (B) Pathways enriched by differentially expressed mRNAs.

The GO functions and KEGG pathway analysis were performed using 99 upregulated and 662 downregulated mRNAs. The mRNAs were enriched in 801 GO‐BP terms, 69 G0‐CC terms, and 64 GP‐MF terms, respectively (Table S8). The top 10 highly enriched GO terms of biological process (BP), cellular component (CC), and molecular function (MF) for mRNAs are shown in Figure 2A. The GO‐BP enrichment result showed that diff‐mRNAs were mainly involved in the extracellular matrix organization, while the collagen‐containing extracellular matrix was the main term for GO‐MF. In addition, in this study, a total of 19 KEGG pathways were involved in CHOL (Table S8). The KEGG pathway analysis of diff‐mRNAs manifested that diff‐mRNAs were mainly enriched in the PI3K‐Akt signaling pathway, calcium signaling pathway, and MAPK signaling pathway (Figure 2B).

### 3.3 lncRNA and circRNA Associated ceRNA Network

**FIGURE 3:**
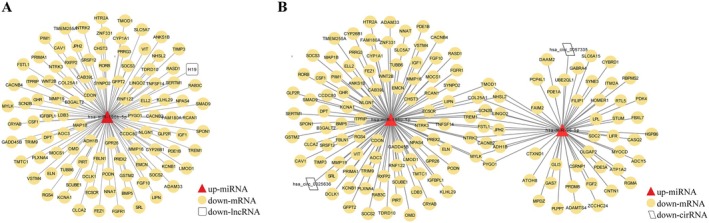
ceRNA network between adjacent cancer and CHOL tissue. (A) LncRNA–miRNA–mRNA regulatory network. (B) CircRNA–miRNA–mRNA regulatory network. The green circle indicates mRNA, the red triangle indicates miRNA, the white quadrilateral indicates lncRNA, and the white parallelogram indicates circRNA.

Regulatory network analysis of lncRNAs, miRNAs, and mRNAs was performed to explore the potential crucial hub lncRNAs involved in the CHOL disease process. Integration results of lncRNAs and miRNAs target gene prediction and co‐expression relationship of diff‐lncRNAs/diff‐mRNAs showed that two lncRNAs (i.e., H19 and DLEU1) and 408 mRNAs with the same trend in expression level were regulated by four same miRNAs (i.e., hsa‐miR‐196b‐5p, hsa‐miR‐320d, hsa‐miR‐106a‐5p, and hsa‐miR‐20b‐5p), and the expression level of these miRNAs was negatively correlated with them. According to the principle that the expression trend between ceRNA and mRNA was consistent but contrary to that of miRNA, the ceRNA regulatory network was re‐screened and sorted out as shown in Figure 3 and Table S9. The lncRNA‐miRNA‐mRNA interactions are shown in Figure 3A. Among them, one down‐lncRNA (i.e., H19), one up‐miRNA (i.e., hsa‐miR‐196b‐5p), and 101 downregulated mRNAs were involved.

Accordingly, regulatory network analysis of circRNAs‐associated ceRNA network between adjacent cancer group and CHOL group was also performed using diff‐circRNAs, diff‐miRNAs, and diff‐mRNAs. The results showed two down‐circRNAs (i.e., hsa_circ_0025636 and hsa_circ_0057335), 154 downregulated mRNAs, and two up‐miRNAs (i.e., hsa‐miR‐96‐5p and hsa‐miR‐196b‐5p) were involved in the circRNA‐miRNA‐mRNA network (Figure 3B).

### 3.4 Protein–Protein Interaction (PPI) Network

**FIGURE 4:**
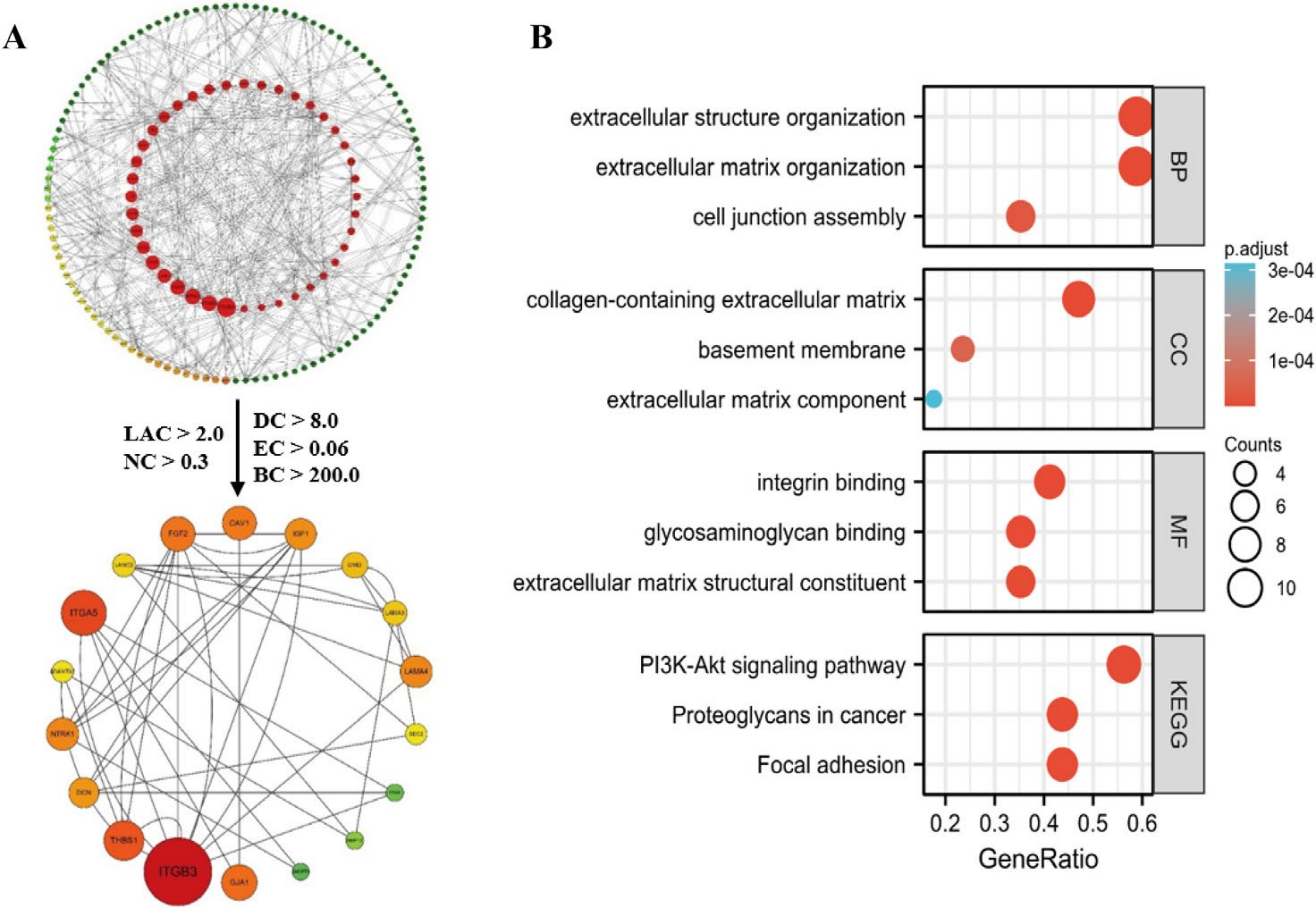
Protein–protein interaction (PPI) Network of core genes and enrichment. (A) Protein–protein interaction (PPI) network. (B) Top 10 Terms enriched by core genes extracted from protein–protein interaction (PPI) network.

The PPI network based on diff‐mRNA consisted of 761 nodes and 103 interaction pairs. The nodes with high topological scores can be regarded as key nodes of the network. There were 62 kinds of interaction relationships among 18 nodes in the network (Figure 4A, Table S10), so the 18 core nodes were identified as core proteins in the process of CHOL occurrence (BC > 200, DC > 8.0, EC > 0.06, LAC > 2.0 and NC > 0.3). Moreover, the genes (*ITGB3, THBS1, DCN, NTRK1, ADAMTS5, LAMC2, ITGA5, FGF2, CAV1, IGF1, LAMA3, DMD, LAMA4, SDC2, FBN1, MMP13, ANGPT1, GJA1*) that meet the criteria were performed for enrichment analysis (Figure 4B). The key genes were significantly involved in hsa04151 ~ PI3K‐Akt signaling pathway (Table S11). Among them, *FGF2, IGF1, CAV1, SPON1*, and *ADAMTS4* are also involved in the ceRNA network in this study.

### 3.5 TCGA CHOL Data Verification

**FIGURE 5:**
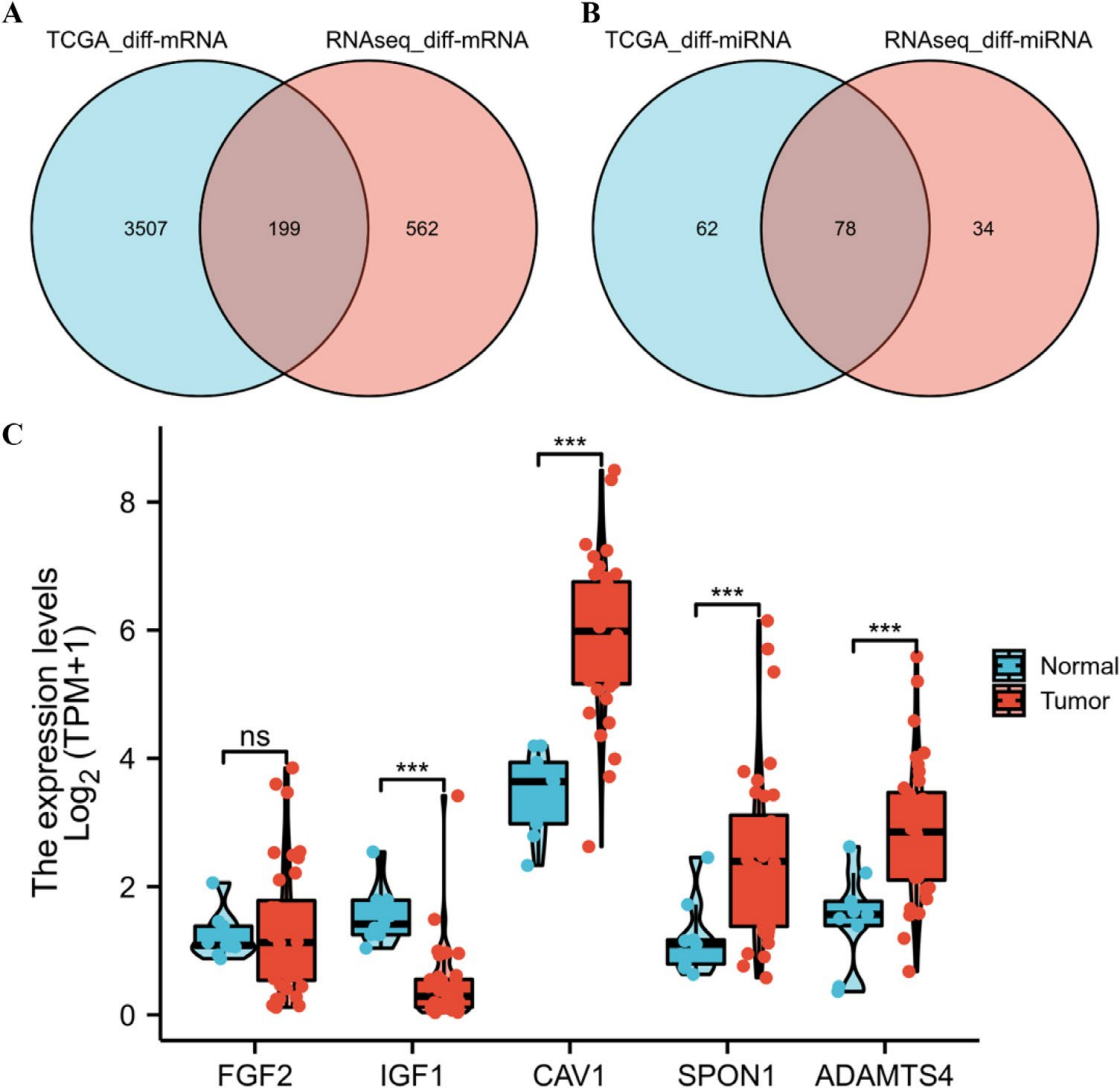
TCGA CHOL data validation. (A) Venn plot of diff‐mRNAs between TCGA‐CHOL and RNAseq. (B) Venn plot of diff‐miRNAs between TCGA‐CHOL and RNAseq. (C) The verification of core genes extracted from protein–protein interaction network using TCGA CHOL data.

According to the threshold (the absolute value of log_2_FoldChange > 2 and the *p* < 0.05), a total of 4805 diff‐mRNAs and 140 diff‐miRNAs were obtained from TCGA‐CHOL. As shown in Figure 5A,B, there were 199 shared diff‐mRNAs and 78 shared diff‐miRNAs in TCGA data and our data. Then, TCGA CHOL mRNA data were obtained to verify the core genes of our sequencing data. Core genes with a higher DC (> 8), EC (> 0.06), LAC (> 2.0), BC(> 200), and NC(> 0.3) in the enriched modules were also expressed differentially in TCGA‐CHOL data (36 tumor samples and 9 adjacent cancer samples), including *FGF2, IGF1, CAV1, SPON1*, *and ADAMTS4* (Figure 5C).

### 3.6 Downregulation of CAV1 Inhibits Proliferation, Migration, and Invasion of Cholangiocarcinoma Cells

**FIGURE 6:**
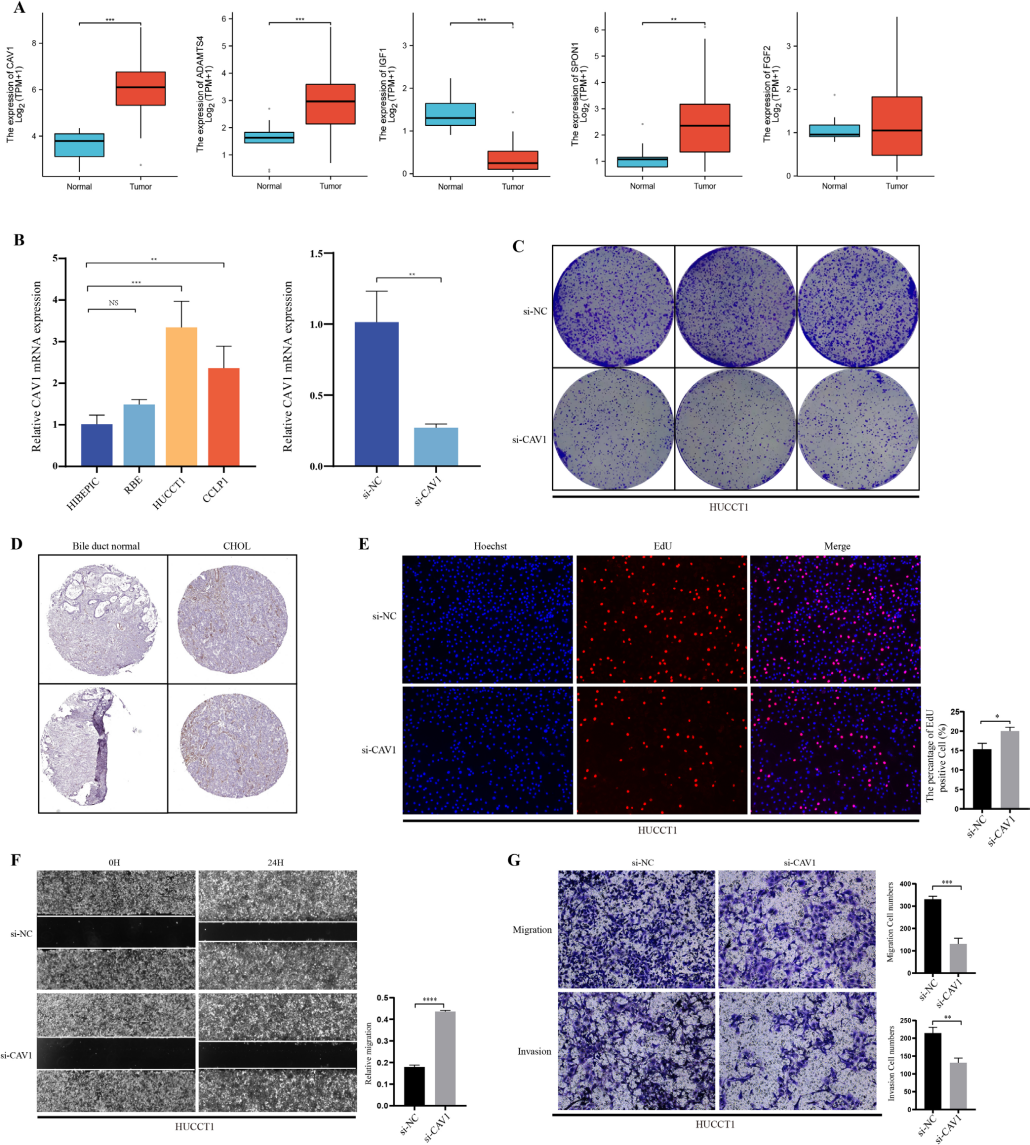
Differential expression and functional validation of CAV1. (A) Relative expression of CAV1 in paired CHOL samples from the TCGA database. (B) Relative expression of CAV1 in cholangiocarcinoma cell lines and knockdown efficiency of si‐CAV1 measured by q‐PCR. (C) Colony formation assay in HUCCT1 cells. (D) Representative immunohistochemical staining of CAV1 in normal biliary tissue and cholangiocarcinoma tissue (HPA049326). (E) EdU proliferation assay comparing proliferation between si‐NC and si‐CAV1 groups. (F) Wound healing assay in HUCCT1 cells. (G) Migration and invasion assays in HUCCT1 cells. *p < 0.05, **p < 0.01.

Based on the diff‐mRNA PPI network, we identified 18 core nodes and obtained 5 key genes, FGF2, IGF1, CAV1, SPON1, and ADAMTS4, that intersect with the ceRNA network. These 5 differential genes were validated for differential expression in the subsequent TCGA‐CHOL cohort, and we analyzed the differential expression levels of each gene (Figure 6A). CAV1 exhibited the most significant differential expression in the analysis. Although previous studies have indicated the importance of CAV1 in the development and progression of cancers in organs such as the colon and breast, its expression and role in cholangiocarcinoma have not been thoroughly explored.

Therefore, we first verified the differential expression levels of CAV1 in cholangiocarcinoma‐related cell lines, which showed varying degrees of elevated expression in cholangiocarcinoma cell lines, with the highest expression level in HUCCT1. Hence, we chose HUCCT1 for further si‐CAV1 transfection studies and verified the knockdown efficiency via qPCR (Figure 6B). Additionally, immunohistochemistry staining images from the Human Protein Atlas (HPA) database showed higher expression levels of CAV1 in cholangiocarcinoma tissues compared to normal tissues (Figure 6D).

We assessed the colony formation ability of cholangiocarcinoma cells after knocking down CAV1 and found a significant reduction in the number of colonies in the si‐CAV1 group compared to the si‐NC group (Figure 6C). Similarly, the EdU proliferation assay indicated that the knockdown of CAV1 significantly inhibited the proliferation of HUCCT1 (Figure 6E). This suggests that downregulation of CAV1 in cholangiocarcinoma cells can significantly inhibit their growth and proliferation. We then evaluated the impact of CAV1 on the migration and invasion of HUCCT1. The scratch assay and Transwell migration assay showed that the migration ability of cells transfected with CAV1 was significantly lower than that of untransfected cells, while the invasion assay revealed that the number of invasive cells in the si‐CAV1 group was significantly lower than that in the si‐NC group. In summary, downregulation of CAV1 expression in cholangiocarcinoma cells can significantly inhibit their proliferation, migration, and invasion abilities.

## 4 Discussion

Most patients with CHOL generally have a dismal prognosis, and surgical resection is not a friendly treatment for advanced patients. Therefore, there is an urgent need to increase the understanding of its molecular tumor biology. Whole transcriptome sequencing technology can provide more information for the treatment and prognosis of CHOL at the transcription level. Our study found that the differential expression of miRNAs, lncRNAs, and circRNAs is closely associated with tumor progression in cholangiocarcinoma. These differentially expressed mRNAs are mainly enriched in the PI3K‐Akt, calcium signaling, and MAPK signaling pathways, which play key roles in tumor cell proliferation and migration. We identified potential key miRNAs in the CHOL disease process, such as hsa‐miR‐196b‐5p. The evidence shows that hsa‐miR‐196b‐5p impedes cell proliferation and gene expression in various cancers such as non‐small cell lung cancer, metastasis in breast cancer, and keloid fibroblast progression. We validated its function in cholangiocarcinoma, consistent with previous studies that suggested miR‐196b‐5p's role in other cancers. Future mechanism studies need to determine the position of hsa‐miR‐196b‐5p in CHOL and its regulated relationship with lncRNA H19. Chu et al. [15] also use whole transcriptome sequencing technology to analyze different genes and constructed a ceRNA network between tissues with eight CHOL patients and tissue adjacent to carcinoma. However, we acquired different diff‐genes and ceRNA networks. The possible reason might be the limited sample size, the tumor heterogeneity, different stages of progression, and different control samples. The difference between adjacent cancer tissue and tumor tissue may not be as large as the difference between normal tissue and tumor tissue. Therefore, we did not find as many differential mRNAs, miRNAs, lncRNAs, and circRNAs as Chu et al. did.

Several reports have been made about lncRNA H19, hsa‐miR‐196b‐5p, and hsa‐miR‐96‐5p in hepatocellular carcinoma or other cancers or tumorigenesis [18, 19, 20, 21, 22, 23]. LncRNA H19 is an imprinted and maternally expressed gene that is conserved in humans and mice and plays a critical role in regulating cell proliferation and differentiation [24]. Studies have shown that cholangiocyte‐derived lncRNA H19 plays a pivotal role in activating hematopoietic stem cells and promoting cholestatic liver fibrosis. H19 is highly expressed in the fetal liver but is significantly inhibited after birth, and the overexpression of lncRNA H19 was also an adaptive mechanism in response to liver cell injury [23]. In addition, several articles showed that hsa‐miR‐196b‐5p was related to multiple sclerosis, prostate cancer, endometriosis, and colorectal cancer [25, 26, 27, 28].

CircRNAs have been confirmed to be a class of covalently closed single‐stranded RNAs formed by reverse splicing of precursor mRNAs [29]. TFPI, as the precursor mRNA of hsa_circ_0057335, has been proved to be closely related to the increased expression of CD33 in CHOL [30] and the decreased expression of TFPI‐2 may inhibit cell migration and tumor invasion, playing an essential role in the carcinogenesis and progression of CHOL. The precursor mRNA of hsa_circ_0025636, RASSF8, has also been predicted as a potential prognostic marker for CHOL [31]. However, there has been no report about hsa_circ_0025636 and hsa_circ_0057335 related to CHOL. We first reported the potentially crucial role of hsa_circ_0025636, hsa_circ_0057335, and hsa‐miR‐196b‐5p in the CHOL disease process.

Studies have shown the role of IGF‐1 and IGF‐1R in regulating tumor cell growth in human cholangiocarcinoma and cholangiocarcinoma cell lines (Huh‐28, TFK‐1, MZ‐CHA‐1). IGF‐1 and IGF‐1R were expressed in all intrahepatic cholangiocarcinoma biopsies. Human intrahepatic cholangiocarcinoma expresses IGF‐1 and IGF‐1 receptors, which act synergistically in regulating cell growth and apoptosis [32, 33]. In addition, CAV1 is an independent positive prognostic factor for survival. A study of the role of CAV1 in 60 patients with extrahepatic cholangiocarcinoma showed that CAV1 was expressed only in tumor cells, but not in normal paracancer tissues [34]. Its expression was negatively correlated with patients' age and tumor size. On this basis, our study constructed the relevant ceRNA network to analyze the regulatory changes in the post‐transcriptional level of gene expression, laying a certain foundation for diagnostic markers of CHOL.

In conclusion, we used whole transcriptome sequencing to reveal the differentially expressed coding and non‐coding RNA profiles in CHOL and constructed a ceRNA network. This study not only highlights the critical roles of lncRNAs, circRNAs, and miRNAs in the molecular regulation of CHOL, but also uncovers previously unreported molecular interactions that may play a key role in tumor progression. This study identified a series of significantly differentially expressed genes and non‐coding RNAs, and further screened core molecules with key regulatory positions in the ceRNA network and protein interaction network. These molecules hold promise as potential serum or histological biomarkers, offering references for early detection and risk stratification. Starting from the transcriptome level, we systematically analyzed the mutual regulation among mRNA, miRNA, lncRNA, and circRNA, providing preliminary insights into their possible “interactive amplification” or “interactive inhibition” mechanisms in cholangiocarcinoma development. In particular, we predicted and discussed the critical roles of non‐coding RNAs (such as lncRNA H19 and circRNA hsa_circ_0057335) in the competitive endogenous RNA (ceRNA) network. The lncRNA‐miRNA‐mRNA and circRNA‐miRNA‐mRNA networks centered on H19, hsa‐miR‐196b‐5p, hsa_circ_0025636, and hsa_circ_0057335 reveal pivotal regulatory axes in the tumorigenesis and progression of cholangiocarcinoma. Targeting these axes could disrupt tumor‐promoting loops. Furthermore, the functional validation of CAV1 as a driver of proliferation, migration, and invasion underscores its potential as a therapeutic target. This provides a theoretical basis for the development of targeted therapeutic strategies aimed at these pathways, offering more effective treatment options for CHOL patients. However, due to the limited sample size and the use of adjacent cancer tissues as controls, this study may not provide a comprehensive understanding of the molecular mechanisms of CHOL at the whole transcriptome level. Furthermore, a larger sample size is required to validate the differentially expressed RNAs and ceRNA networks identified in this study.

## Author Contributions

**Qinlei Wang:** formal analysis (equal), methodology (equal), software (equal), validation (equal), visualization (equal), writing – original draft (equal), writing – review and editing (equal). **Menshou Chen:** formal analysis (equal), methodology (equal), validation (equal). **Jingru Zhang:** formal analysis (equal), methodology (equal). **Chuan Feng:** conceptualization (equal), methodology (equal), supervision (equal), validation (equal), visualization (equal), writing – review and editing (equal). **Haoran Li:** methodology (equal). **Jingyu Guo:** formal analysis (equal), writing – original draft (equal). **Zhaowei Sun:** conceptualization (equal), data curation (equal), methodology (equal), validation (equal), writing – original draft (equal). **Yujie Feng:** formal analysis (equal), funding acquisition (equal), project administration (equal), supervision (equal), validation (equal), visualization (equal).

## Ethics Statement

The studies involving human participants were reviewed and approved by the Ethics Committee of the Affiliated Hospital of Qingdao University (approval no. QYFYWZLL26664). The purpose, along with the methods of this research, were fully disclosed to all eligible patients.

## Consent

The patients/participants provided their written informed consent to participate in this study. The study is conducted in accordance with the Helsinki Declaration, the International Conference of Harmonization Good Clinical Practice guidelines, and local regulatory requirements.

## Conflicts of Interest

The authors declare no conflicts of interest.

## Supporting information


**Table S1.** Clinical characteristics of six patients.
**Table S2.** The primer sequences used in this study.
**Table S3.** Differentially expressed mRNAs between adjacent cancer group and cancer group in CHOL.
**Table S4.** Differentially expressed miRNAs between adjacent cancer group and cancer group in CHOL.
**Table S5.** Differentially expressed lncRNAs between adjacent cancer group and cancer group in CHOL.
**Table S6.** Differentially expressed circRNAs between adjacent cancer group and cancer group in CHOL.
**Table S7.** Comparison of differential expressed RNAs between similar studies and our research.
**Table S8.** The GO functions and KEGG pathway of diff‐mRNAs between adjacent cancer tissues and cancer groups in CHOL.
**Table S9.** LncRNA/CircRNA–miRNA–mRNA regulatory networks between adjacent cancer tissues and cancer group in CHOL.
**Table S10.** Protein–Protein Interaction (PPI) Network and Core Proteins Extraction from diff‐mRNAs of CHOL.
**Table S11.** The GO functions and KEGG pathway of core proteins between adjacent cancer tissues and cancer groups in CHOL.

## Data Availability

The raw data of whole transcriptome sequencing reported in this study were archived in the Sequence Read Archive (SRA) of NCBI with the accession number PRJNA769618.
